# Supplementary material for: Polyketide synthase gene clusters in the ericoid mycorrhizal fungus Oidiodendron maius: genome-wide prediction, expression profiling, and functional characterization of knockout mutants
Source: Front Plant Sci. 2026 Sep 16;17:1924430. doi: 10.3389/fpls.2026.1924430 (PMC13623903; doi:10.3389/fpls.2026.1924430)
Supplement: Supplementary file 1 [file SupplementaryFile1.zip › Supplementary Materials/Supplementary Folder 1.zip!/Oidma1_AssemblyScaffolds_Repeatmasked/knownclusterblast/cluster54/jgi.p_Oidma1_184951_mibig_hits.html]

| MIBiG Protein | Description | MIBiG Cluster | MiBiG Product | % ID | % Coverage | BLAST Score | E-value |
| --- | --- | --- | --- | --- | --- | --- | --- |
| OAQ83755.1 | isotrichodermin\_C-15\_hydroxylase | BGC0001358 | Leucinostatins\_biosynthetic\_gene\_cluster | 33 | 96.1240310078 | 280 | 2.9e-75 |
| AAC49200.1 | putative\_p450\_monooxygenase | BGC0000152 | Sterigmatocystin\_biosynthetic\_gene\_cluster | 34 | 95.9302325581 | 261 | 1.8e-69 |
| ACH72905.1 | AflG | BGC0000011 | Aflatoxin/sterigmatocystin\_biosynthetic\_gene\_cluster | 34 | 91.2790697674 | 257 | 4.5e-68 |
| AAC49196.1 | putative\_p450\_monooxygenase | BGC0000152 | Sterigmatocystin\_biosynthetic\_gene\_cluster | 33 | 91.2790697674 | 255 | 1.7e-67 |
| ACD39751.1 | cytochrome\_P450 | BGC0000076 | Hypothemycin\_biosynthetic\_gene\_cluster | 33 | 94.5736434109 | 251 | 2.5e-66 |
| ACD39760.1 | cytochrome\_P450 | BGC0000077 | Hypothemycin\_biosynthetic\_gene\_cluster | 33 | 94.5736434109 | 250 | 5.5e-66 |
| AAM18769.1 | AflL | BGC0000011 | Aflatoxin/sterigmatocystin\_biosynthetic\_gene\_cluster | 33 | 95.7364341085 | 248 | 1.2e-65 |
| BAD83682.1 | cytochrome\_P-450 | BGC0000012 | Alternapyrone\_biosynthetic\_gene\_cluster | 33 | 98.4496124031 | 247 | 2.7e-65 |
| AAS90079.1 | VerB | BGC0000010 | Aflatoxin\_biosynthetic\_gene\_cluster | 34 | 96.8992248062 | 247 | 4.6e-65 |
| AAS90102.1 | VerB | BGC0000006 | Aflatoxin\_biosynthetic\_gene\_cluster | 34 | 96.8992248062 | 247 | 4.6e-65 |
| BAE71326.1 | desaturase | BGC0000004 | Aflatoxin\_biosynthetic\_gene\_cluster | 34 | 97.0930232558 | 247 | 4.6e-65 |
| AAS90033.1 | VerB | BGC0000008 | Aflatoxin\_biosynthetic\_gene\_cluster | 34 | 96.8992248062 | 246 | 6e-65 |
| AAS90011.1 | VerB | BGC0000007 | Aflatoxin\_biosynthetic\_gene\_cluster | 33 | 97.6744186047 | 244 | 2.3e-64 |
| AAS90059.1 | VerB | BGC0000009 | Aflatoxin\_biosynthetic\_gene\_cluster | 34 | 97.8682170543 | 243 | 5.1e-64 |
| XP\_002384857.1 | cytochrome\_P450,\_putative | BGC0001234\_c2 | 4,4'-piperazine-2,5-diyldimethyl-bis-phenol\_biosynthetic\_gene | 31 | 99.0310077519 | 242 | 8.7e-64 |
| ACB12551.1 | Fum2 | BGC0000063 | Fumonisin\_biosynthetic\_gene\_cluster | 32 | 93.992248062 | 237 | 2.8e-62 |
| AAN74815.2 | Fum2p | BGC0000062 | Fumonisin\_biosynthetic\_gene\_cluster | 31 | 97.480620155 | 234 | 3.1e-61 |
| BAD83680.1 | cytochrome\_P-450 | BGC0000012 | Alternapyrone\_biosynthetic\_gene\_cluster | 30 | 101.162790698 | 233 | 5.3e-61 |
| BAK26559.1 | cytochrome\_P450 | BGC0000977 | Cyclopiazonic\_acid\_biosynthetic\_gene\_cluster | 31 | 98.0620155039 | 225 | 1.1e-58 |
| ACD39773.1 | cytochrome\_P450 | BGC0000134 | Radicicol\_biosynthetic\_gene\_cluster | 33 | 96.7054263566 | 224 | 2.5e-58 |
| ADI24937.1 | VrtK | BGC0000168 | Viridicatumtoxin\_biosynthetic\_gene\_cluster | 30 | 97.6744186047 | 222 | 1.6e-57 |
| BAD29972.1 | P450\_monooxygenase\_1 | BGC0000676 | Aphidicolin\_biosynthetic\_gene\_cluster | 31 | 98.2558139535 | 220 | 4.6e-57 |
| BAE56591.1 |  | BGC0001123 | Aspirochlorine\_biosynthetic\_gene\_cluster | 31 | 96.1240310078 | 220 | 6.1e-57 |
| Bcin12g06370.1 |  | BGC0000631 | Botrydial\_biosynthetic\_gene\_cluster | 27 | 96.3178294574 | 169 | 9.4e-42 |
| ADI24958.2 | GsfF | BGC0000070 | Griseofulvin\_biosynthetic\_gene\_cluster | 25 | 95.3488372093 | 125 | 2e-28 |
| AAC49192.1 | putative\_p450\_monooxygenase | BGC0000152 | Sterigmatocystin\_biosynthetic\_gene\_cluster | 30 | 36.0465116279 | 88 | 2.1e-17 |
| CYP719A21 |  | BGC0001325 | Noscapine\_biosynthetic\_gene\_cluster. | 26 | 37.984496124 | 82 | 1.2e-15 |
| ALA62324.1 | methylphloroacetophenone\_synthase | BGC0001309\_c2 | Usnic\_acid\_biosynthetic\_gene\_cluster | 27 | 36.8217054264 | 57 | 4e-08 |
| ctg1\_orf04 |  | BGC0000685 | Brassicicene\_C\_biosynthetic\_gene\_cluster | 39 | 20.5426356589 | 56 | 8.9e-08 |
| ADM79460.1 | P450\_protein | BGC0001266 | Grayanic\_acid\_biosynthetic\_gene\_cluster | 24 | 39.5348837209 | 51 | 2.9e-06 |
